# On the Role of Electronic Correlation and State‐Specific Environment Polarization in Singlet–Triplet Gap Inversion

**DOI:** 10.1002/jcc.70267

**Published:** 2025-11-11

**Authors:** Ester Salvi, Giacomo Agostini, Simone Veglianti, Gustavo Juliani Costa, Luca De Vico, Daniele Padula, Ciro A. Guido

**Affiliations:** ^1^ Dipartimento di Scienze e Innovazione Tecnologica Università del Piemonte Orientale Alessandria Italy; ^2^ Dipartimento di Biotecnologie Chimica e Farmacia, Università di Siena Siena Italy

**Keywords:** double‐hybrid density functional theory, electronic correlation, environment polarization, excited states, multireference electronic structure, singlet–triplet inversion, state specific solvation, TDDFT, thermally activated delayed fluorescence (TADF)

## Abstract

Molecules characterized by an inverted singlet‐triplet gap ($\Delta E_{\mathrm{ST}}<0$) hold potential for optoelectronic applications. Electronic correlation and environmental polarization are key factors influencing negative $\Delta E_{\mathrm{ST}}$, and the latter is gaining attention for its possible role in “mimicking” correlation contributions to yield negative $\Delta E_{\mathrm{ST}}$. However, a comprehensive study of solvation effects on both structures and energy gaps is still lacking. In this work, we evaluate computational strategies for calculating $\Delta E_{\mathrm{ST}}<0$ gaps, incorporating electronic correlation and solvent polarization in molecules exhibiting singlet‐triplet inversion. Using RMS–CASPT2 as a benchmark, we demonstrate that double‐hybrid density functionals and mixed‐reference spin‐flip TD‐DFT (MRSF–TD‐DFT) can partially recover electronic correlation. Furthermore, we investigate solvation effects on both singlet and triplet excited states, highlighting the limitations of linear‐response schemes in continuum solvation models. We finally develop a protocol combining electronic correlation and state‐specific solvent polarization using double‐hybrid functionals and the Vertical Excitation Model (VEM), leveraging its Lagrangian implementation to compute structures and adiabatic energies. Applying our B2PLYP/VEM(UD) protocol to larger systems with experimentally observed negative $\Delta E_{\mathrm{ST}}$ gaps, we quantitatively reproduce experimental emissive and non‐radiative transition rates.

## Graphical abstract

Dynamic electronic correlation and state‐specific solvent polarization act cooperatively to invert the singlet–triplet energy gap in organic emitters. The B2PLYP/VEM(UD) protocol combines double‐hybrid functionals with the state‐specific Vertical Excitation Model and quantitatively reproduces experimental adiabatic inverted gaps and emission rates, offering valuable insights for the design of next‐generation TADF and INVEST materials.

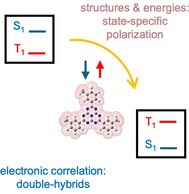

## 1 Introduction

In recent years, molecules where the first triplet electronic state lies energetically above the first singlet excited state (known as IST or INVEST emitters) have emerged as promising candidates for optoelectronic applications [1, 2, 3]. Although known since the 1980s [4, 5, 6, 7, 8], IST emitters' technological relevance has only recently been recognized [2, 3], mainly due to the renewed interest in thermally activated delayed fluorescence (TADF), a phenomenon known since the 1960s [9] in the realm of Organic Light Emitting Diodes (OLEDs) [10, 11, 12]. IST substrates with significant fluorescence rates could surpass TADF–based emitters by facilitating a rapid, non‐activated reverse intersystem crossing process (rISC) [13]. This capability is crucial for contrasting the inevitable formation of triplet states resulting from charge recombination [14], and for enhancing efficiency without relying on costly and potentially hazardous metals.

To improve currently known materials and design novel molecules, accurate simulation tools are key. Since the resurgence of interest in these substrates, electronic correlation has become evident as a key factor for their accurate description [2, 3]. In recent years, many studies [2, 15, 16] pointed out that Configuration Interaction Singles (CIS) cannot yield negative $\Delta E_{\mathrm{ST}}$, since the difference between the singlet and triplet Hamiltonian matrix is twice the matrix of two‐electron Coulombic integrals (positive semi‐definite) [2, 17], highlighting the crucial role of correlation effects, such as including doubly excited configurations, when necessary, between frontier orbitals [18]. However, even if this effect can by itself drive singlet–triplet gap inversion, a balanced inclusion of dynamic correlation is required for quantitative accuracy [19]. The different spatial distribution of frontier molecular orbitals involved in the electronic transition of many TADF emitters has been extensively investigated [20, 21] and can be assessed using diagnostic tools for the widely‐used time‐dependent density functional theory (TD‐DFT) [22]. In this regard, a particularly useful parameter is Δr [23], which describes the average hole–electron distance upon excitation, as some of us have previously demonstrated in a screening scenario [24]. It was also shown that methods incapable of describing conical intersections fail to accurately predict singlet–triplet inversion [2]. While TD‐DFT can theoretically yield negative singlet–triplet gap values [2, 25], in practice, the correlation contribution is negligible compared to the Coulomb interactions. Indeed, the singlet–triplet matrix difference tends to remain positive definite for the typically used exchange–correlation (xc) functionals, owing to the prevalent adiabatic approximation with its frequency‐independent kernel: the adiabatic xc‐contribution is negative for excitations dominated by a single transition [2, 16, 22, 26], and in principle, a sufficiently strong correlation contribution could reduce or even invert the singlet–triplet gap.

Apart from predicting $\Delta E_{\mathrm{ST}}$ gap values through reliable theoretical methods and geometries [27, 28, 29, 30], it is essential to emphasize the central role played by crossing points between potential energy surfaces (PESs) in this application. It is crucial that the $S_{1}$/$T_{1}$ minimum energy crossing point (MECP), regulating the ISC and rISC processes, is accessible from the $T_{1}$ minimum through thermal fluctuations. Once the $S_{1}$ state is populated, it is necessary to avoid $S_{1}$/$S_{0}$ conical intersections to, instead, promote radiative relaxation back to $S_{0}$. Although several studies focused on the rISC process, the identification of $S_{1}$/$S_{0}$ minimum energy conical intersections (MECI)s has not attracted the community's interest until very recently [28].

Alongside electronic correlation, the influence of the environment is now receiving growing attention. Recent work [31] indicates that solvent polarization seems to “mimic” additional correlation contributions to produce negative $\Delta E_{\mathrm{ST}}$ values. However, evidence of the effect of the environment on molecules exhibiting singlet‐triplet inversion has so far only been presented with ΔDFT methods [32], and a comprehensive study of the role of solvation models on both structures and energy gaps at the TD‐DFT level has not yet been conducted. It is crucial to include the different characteristic response times of chromophores and environment (such as non‐equilibrium effects) and a diverse description of the medium polarization response to excitations [33] when simulating environmental effects on the formation and relaxation of electronic excited states. Previous studies on singlet excited states have highlighted the importance of incorporating state‐specific (SS) corrections to the conventional linear response (LR) framework of polarizable continuum models (LR‐PCM) when a significant reorganization of the electron density occurs during electronic excitations, specifically to recover the complete response of the environment polarization [33, 34, 35].

In this work, we aim to (i) benchmark $\Delta E_{\mathrm{ST}}$ gaps with methods capable of identifying PES crossing points, (ii) assess the impact of dynamic correlation and state‐specific solvent embedding on $\Delta E_{\mathrm{ST}}$ gaps and excited‐state structures, and (iii) apply these methods to substrates unsuitable for conventional tools yet promising as dyes for devices. To achieve the first two objectives, we compute adiabatic $\Delta E_{\mathrm{ST}}$ gaps on a benchmark set of small molecules exhibiting singlet–triplet inversion. We exploit different multiconfigurational techniques, including second–order perturbative corrections, to take into account both static and dynamic correlation effects. In particular, we adopted the RMS–CASPT2 method [36, 37], and the mixed–reference spin–flip variant of TD‐DFT (MRSF–TD‐DFT) [38, 39, 40, 41, 42, 43]. The latter overcomes spin contamination and single‐excitation limitations intrinsic to (spin‐flip) density functional theory [44, 45], and it has been recently applied to multiresonance TADF materials [46], making the application to IST systems the next step challenge for this method. We finally present a computational strategy to treat the first singlet and triplet excited‐state energies of solvated IST systems, to capture contributions resulting from interactions with the environment. Our protocol is based on the application of the vertical excitation method (VEM) [47], which is a state‐specific, self‐consistent, and variational approach to solvent polarization response. Therefore, energies, structures, and properties can be computed in a state‐specific solvation framework [48]. The solvent model must be coupled with a correspondingly reliable electronic structure description: we therefore provide a definition for the $S_{1}$ and $T_{1}$ adiabatic energies that contains a second‐order perturbative correction, derived from double–hybrid TD‐DFT/B2PLYP calculations, applied to state specific excitation energies. Initially benchmarked on five small substrates, this computational procedure has been applied to large systems of experimental relevance.

## 2 Methodology

We obtained equilibrium geometries of $S_{0}$, $S_{1}$, and $T_{1}$ electronic states at DFT and TD‐DFT level using the M06–2X functional [49] with the def2–TZVP basis set [50], since several examples from the literature demonstrate reliability of these geometries [24, 51, 52]. All these simulations have been performed employing the Gaussian16 software [53]. To ensure reliability of the adopted geometries, we also carried out $S_{1}$ optimizations at RMS–CASPT2/cc–pVDZ level for molecules **1–4**, reporting in Table S1 the root mean square deviations between TDDFT and RMS–CASPT2 geometries.

Vertical (i.e., at the equilibrium geometry of the ground state) and adiabatic (i.e., at the minimum of each state) $\Delta E_{\mathrm{ST}}$ gaps have been computed adopting different electronic structure methods. We performed multireference calculations at the RMS–CASPT2 level [36, 37] with the triple‐ζ ANO–R2 basis set [54] on top of SA(5)–CASSCF wavefunctions, running single point calculations on the geometries obtained as previously described. We used five states in the state‐averaging procedure to maintain consistency with previous results [16, 24, 55, 56], and to be sure to include a number of states that, after potential reordering due to perturbative corrections, would yield the correct $S_{1}$ and $T_{1}$. RMS–CASPT2 calculations were performed both in vacuo and in solvent, using the conductor‐like version of PCM (CPCM) [57, 58], as implemented in OpenMolcas [59]. We note that the coupling of multireference electronic structure approaches with PCM is intrinsically a state–specific implementation of solvation [60, 61], since the reaction field of each electronic state originates from the wavefunction of the state of interest. To provide a qualitative rationale for the computed $\Delta E_{\mathrm{ST}}$ values, the HOMO–LUMO exchange integral $K_{\mathrm{HL}}$ is also calculated. Within single‐reference methods such as CIS or TD‐DFT, the singlet–triplet energy gap, when mainly arising from a HOMO → LUMO transition, can approximately be estimated as twice $K_{\mathrm{HL}}$. This simple relationship makes $K_{\mathrm{HL}}$ a useful diagnostic for interpreting trends in a qualitative way [2, 15, 17, 24, 62]. We computed this quantity from SA(5)–CASSCF Natural Orbitals (NOs) with the Multiwfn software [63]. The HONO was chosen as the least occupied active space orbital in the set of those doubly occupied in the SCF configuration, and the LUNO as the most occupied active orbital in the set of those empty in the SCF configuration.

We carried out MRSF–TD‐DFT [39] calculations of adiabatic $\Delta E_{\mathrm{ST}}$ gaps with the GAMESS software [64], using the def2–TZVP basis set [50] in combination with several density functionals. Although MRSF–TD‐DFT is commonly used in combination with BH&HLYP [45], we also adopted a set of other functionals commonly used to study excited states, namely M06–2X [49], PBE0 [65], CAM–B3LYP [66], and the double hybrid B2PLYP [67]. Solvent effects have been included within the CPCM formalism: since the implementation does not include state–specific effects, the LR‐PCM framework has been exploited. $\Delta E_{\mathrm{ST}}$ gaps were computed using energies of the second response state for $S_{1}$ and of the first response state for $T_{1}$, ignoring the energy of the reference triplet, which is obtained variationally [38, 68]. For the sake of comparison, we also performed TD‐DFT simulations using the same functionals, in the standard RPA (also known as Casida) formulation. State–specific solvation at the TD‐DFT level has been included, exploiting the VEM approach [47] in its Lagrangian formulation [48], which includes analytical gradients. The VEM solvent polarization response scheme, whose full formulation is detailed in Refs. [47, 48], is self‐consistent, as it evaluates the excited‐state reaction field iteratively, that is, in a manner consistent with either the relaxed (RD) or unrelaxed (UD) density difference between the ground and excited states. The approach is state‐specific, as it explicitly accounts for changes in the solvent's reaction field in response to the solute's electronic excitation. This is achieved by using the excited‐state electronic density to calculate the fast polarization contribution to the excitation free energy. Tretiak and coworkers proved that the UD variant is variational [69] and Guido and coworkers developed a Lagrangian formulation at TD‐DFT level of theory [33]. It is important to point out that even if the Lagrangian formulation is developed on the VEM(UD) excited state energy, the gradient of this energy includes also the relaxed density contribution, because it explicitly computes the Z‐vector term, which corresponds to solving the coupled perturbed equations [33]. We therefore determined both excited state energies and geometries, including state–specific polarization response effects. The double hybrid VEM(UD) protocol requires two steps, both carried out with a locally modified version of the G16 software: we first calculated B2PLYP second order perturbatively corrected energies, scaled by 0.27 as in the implementation from Grimme et al. [67]. This term is based on integrals involving molecular orbitals (MOs) from ground state PCM calculations in a CIS(D)‐like implementation, as detailed in Ref. [70]. The VEM(UD) correction to these values is then computed with a modified BLYP functional that contains 53% of exact exchange and added to the former term, since the VEM(UD) variational procedure does not affect ground state MOs.

Finally, vibronic effects on fluorescence and nonradiative rates (i.e., internal conversion (IC), ISC, and rISC) were included using the FCClasses3 program [71]. Since the VEM–UD energy Hessian is unavailable, state‐specific polarization response energies and transition dipoles have been used on top of excited state frequencies and normal modes from LR–PCM calculations. Although it is well known that Herzberg–Teller effects can significantly enhance (up to 2 orders of magnitude) ISC and rISC rates [72, 73], we only included Franck–Condon effects in our calculations: we found that the large singlet‐triplet energy splitting was, by itself, a sufficient driving force for the processes, even in the presence of a low electronic spin‐orbit coupling constant.

## 3 Results

**FIGURE 1:**
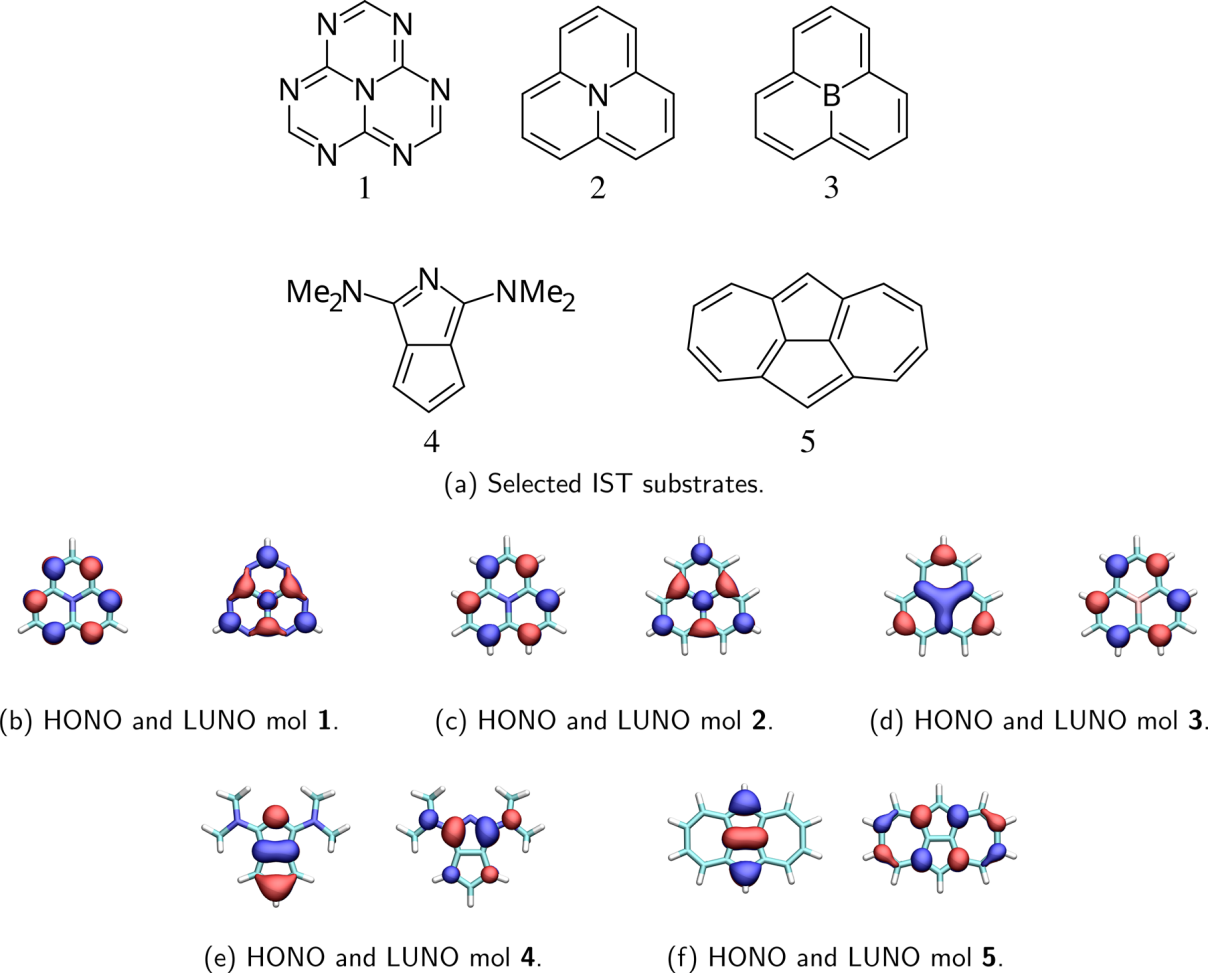
Selected IST substrates and their respective frontier natural orbitals.

The suitability of the computational strategies for addressing IST dyes has been evaluated on five substrates presented in Figure 1a. Molecules **1–3** belong to the triangulene family and **1–2**, i.e., heptazine and cyclazine, are the molecules that, in 2019, set off the rebirth of this field, and as such the object of several benchmarks [29, 74]. Molecules **4–5** contain the pentalene core, a chromophore known to give rise to singlet–triplet inversion since the 1980s [6]. Molecule **4** was recently identified as an IST dye by some of us [24], while molecule **5**, isopyrene, has been known since the 80s [7, 8] and has been the object of a few recent studies [24, 75, 76, 77].

Both $S_{1}$ and $T_{1}$ states of all systems are dominated by a HOMO → LUMO transition, with frontier natural orbitals as depicted in Figure 1b–f. These transitions have a $\pi \to \pi^{*}$ character, with dark $S_{1}$ since they are symmetry‐forbidden. The small spatial superposition (due to the alternating nodal plane symmetry) between frontier orbitals points to a small Coulombic contribution in the excited state [24], remarking the necessity to include correlation effects to predict a negative $\Delta E_{\mathrm{ST}}$. To avoid confusion, it is essential to note that in the RPA approach to TDHF/TDDFT, the response of the Coulomb operator corresponds to an exchange‐like integral (and *vice versa*) [78]. Therefore, the corresponding literature on computational studies applied to TADF refers to it as an *exchange integral* between frontiers orbitals [2]. For the selected substrates, values (reported in Table 1a as $K_{\mathrm{HL}}$) are ≈0.13 eV, in agreement with expectations and past studies adopting CIS. Molecule **4** is the exception, showing a value that is almost double that of the others, but still rather low compared to typical organic systems [24].

**TABLE 1:** Adiabatic ΔE gap (meV) computed using different electronic structure methods.

(a) RMS‐CASPT2/SA(5)‐CASSCF/ANO–R2				
Molecule	Active space	SA(5)–CASSCF	RMS–CASPT2	($K_{\mathrm{HL}}$)^a^
1	(14, 13)	−578	−199	129
2	(14, 13)	−440	−55	131
3	(12, 13)	−412	−143	128
4	(14, 11)	−431	−30	265
5	(16, 16)	−369	−44	120

(b) LR‐TDDFT/def2‐TZVP					
Molecule	Functional				
	BH&HLYP	M06–2X	PBE0	CAM–B3LYP	B2PLYP
1	310	201	231	236	−36
2	297	194	240	262	75
3	271	184	210	240	35
4	360	236	297	302	−5
5	222	138	195	189	−374

(c) MRSF‐TDDFT/def2‐TZVP					
Molecule	Functional				
	BH&HLYP	M06‐2X	PBE0	CAM–B3LYP	B2PLYP
1	−117	−163	4	−73	−139
2	−71	−98	16	−58	−87
3	−98	−145	5	−64	−116
4	110	92	82	91	112
5	−37	−68	20	−17	−49

^a^ $K_{\mathrm{HL}}$ refers to the exchange integral between frontier natural orbitals HONO and LUNO, in meV.

### 3.1 Benchmarking TD–DFT and MRSF–TD‐DFT Against RMS–CASPT2

Let us start the discussion on electronic structure methods from RMS–CASPT2/ANO–R2 simulations of adiabatic gaps, reported in Table 1a, that constitute the reference for our benchmark. For each of the considered molecules, we selected a tailored active space, for which we converged the zeroth–order wavefunction through SA(5)–CASSCF/ANO–R2 calculations before applying perturbative corrections. Molecules **1–5** are all known IST dyes, and both SA–CASSCF and RMS–CASPT2 provided $\Delta E_{\mathrm{ST}}<0$ values, in line with literature data [16, 24, 29, 51, 75]. We note that the inclusion of dynamic correlation effects via second‐order perturbation theory on top of a CASSCF calculation reduces the absolute value of the gap. This is due to the larger stabilization of triplet states with respect to singlet states by the PT2 term (data not shown).

We point out that, although theoretical best estimates computed exploiting single–reference methods such as CCSD(T) or even ADC(2) provide larger negative $\Delta E_{\mathrm{ST}}$ gaps for heptazine and cyclazine in comparison to CASPT2 results [19, 29, 74], all of the few experimental data available in literature show $-0.100<\Delta E_{\mathrm{ST}}<-0.010$ eV [55, 79, 80, 81, 82], highlighting a reasonable performance of CASPT2 in determining such gaps.

In line with assessments from earlier studies [2, 3, 16, 25], LR–TD‐DFT calculations based on single hybrid functionals are unable to provide the negative gaps as determined by multireference simulations (see Table 1b) for all five substrates (see also Tables S2, S3, and S5). Incidentally, this also confirms the criterion of $\Delta E_{\mathrm{ST}}^{\mathrm{TD}‐\mathrm{DFT}}\leq 0.5$ eV, recently introduced by some of us [24], to screen for potential $\Delta E_{\mathrm{ST}}$ inversion using TD–DFT, unable to predict inversion. Importantly, inclusion of dynamic correlation effects using the semi‐empirical double hybrid functional B2PLYP [67] makes it possible to predict negative gaps for molecules **1**, **4**, and **5**. Our results align with the work of Sancho‐Garcia et al. [83] who reported at the same computational level, a vertical ΔE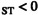 for heptazine and a vertical ΔE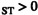 for cyclazine. System **5** also showed a negative ST gap at the double‐hybrid TD‐DFT level in previous works [76]. B2PLYP will therefore be used to perform our TD‐DFT/VEM(UD) simulations in the following.

Finally, we performed MRSF–TD‐DFT calculations to include static correlation effects at TD‐DFT level [55, 56, 84]. Table 1c reports the ΔE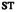 results obtained with different xc‐functionals. Our simulations show that the impact of static correlation is indeed significant: all adopted functionals, except PBE0, provide negative values for substrates **1**, **2**, **3**, and **5**. These results hint at a predominant role of static rather than dynamic correlation, the latter mainly included when adopting a double hybrid functionals such as B2PLYP. This is consistent with recent studies: for instance, building on the mechanism originally proposed by Kollmar and Staemmler [18], Drwal et al. [19] showed that in heptazine derivatives the singlet–triplet gap inversion is primarily driven by dynamic spin‐polarization arising from coupling with doubly excited $\pi -\pi^{*}$ configurations, yet a quantitative description requires the additional inclusion of dynamic correlation to compensate for the overestimation of the inversion induced by spin polarization alone. The trends observed at the RMS–CASPT2 level are quantitatively reproduced, with Pearson's correlation coefficients greater than 0.65. Among the chosen functionals, M06–2X yields the best result (r≈0.78), while BH&HLYP (r≈0.74) and B2PLYP (r≈0.75) produce comparable outcomes. This is in agreement with what was observed in the literature for MRSF–TD‐DFT: functionals including ≈50% of HF exchange are in general recommended [45], as it is the case for BH&HLYP and M06–2X.

To summarize the data presented so far, our methodological assessment, even if performed on a limited number of compounds, supports the idea that MRSF–TD‐DFT is a promising and computationally affordable method for studying these systems. Unlike other popular approaches applied to similar substrates, it accurately captures the multireference nature of wavefunctions at crossing points [41, 85], which are crucial in governing rISC and radiationless decay, thereby playing a key role in the photophysics and photochemistry of these chromophores.

### 3.2 Polarization Response of the Environment: A State‐Specific Description of Structure and Energies

Spectroscopic parameters are likewise greatly influenced by the presence of an embedding. In the case of dyes that constitute the emissive layer of OLEDs, the environment is weakly polar and can be computationally reproduced by a solvent with a dielectric constant ϵ<5 [86, 87]. The main solute‐solvent interactions are of electrostatic origin, and this explains the widespread use of continuum dielectric models, such as PCM [88], for their description. In the context of TD‐DFT calculations, choosing an appropriate continuum model polarization scheme is crucial to correctly reproduce chromophores' properties.

From a theoretical perspective, we can formally think of the excitation in solution as a two‐step process: in the first step, the molecule, initially in its electronic ground state (GS) in the presence of the solvent whose source of polarization is the GS electron density, is excited to the *I*th state with the solvent polarization frozen in the initial ground state. The excitation energy of this formal step is usually indicated as $\omega_{0}^{\left(0I\right)}$. In a second formal step, the dynamic component of the reaction field due to the solvent polarization is considered. Two main formulations have been proposed to describe this component of the solvent response, providing different expressions of the excitation energy of a chromophore in a solvent [33]:

(1) $$\Delta E_{\mathrm{LR}}^{\left(0I\right)}=\omega_{0}^{\left(0I\right)}+R_{\text{solv}}\left(P_{\text{trans}}^{\left(0I\right)}\right)$$

(2) $$\Delta E_{\mathrm{SS}}^{\left(0I\right)}=\omega_{0}^{\left(0I\right)}+R_{\text{solv}}\left(P_{\Delta }^{\left(0I\right)}\right)$$

In a state‐specific (SS) description (Equation 2), the response of the solvent dynamic polarization (i.e., $R_{\text{solv}}$) is a functional of the difference of the electron densities of the initial and final states ($P_{\Delta }^{\left(0I\right)}=P^{I}-P^{0}$) [23]. On the other hand, in the LR‐solvation formulation (Equation 1), the response of the solvent is based on the electron transition density ($P_{\text{trans}}^{\left(0I\right)}$). For chromophores whose GS is a singlet, it follows that this model does not correctly describe the solvation effects on triplet excited states, since $P_{\text{trans}}^{\left(0I\right)}=0$ for a singlet‐to‐triplet transition.

**FIGURE 2:**
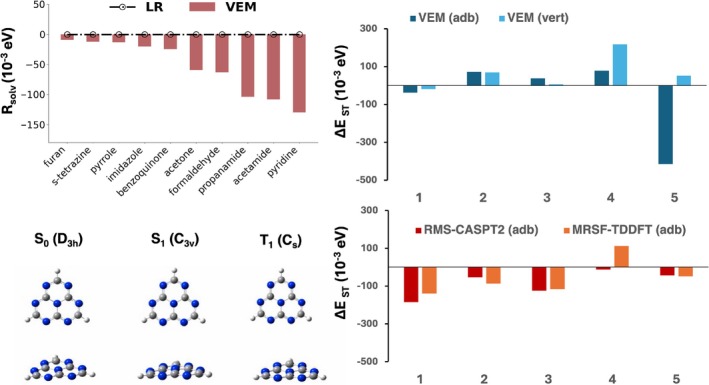
Top, left: LR and VEM solvent polarization response to $T_{1}$ transitions of different molecular systems in toluene (M06‐2X/6‐31+G*); Top, right B2PLYP/VEM(UD) adiabatic and vertical $\Delta E_{\mathrm{ST}}$; bottom, right: RMS‐CASPT2/ANO‐R2 SS‐PCM $\Delta E_{\mathrm{ST}}$ bottom, left: M06‐2X/def2‐TZVP optimized structures of **1** at different point symmetries.

This theoretical limitation was quantitatively assessed by comparing TD‐DFT LR‐PCM and the VEM(UD) (i.e., a SS‐PCM approach) $S_{0}\to T_{1}$ excitation energies simulations performed on a set of systems already investigated for the benchmarking of triplet excited states calculations in previous studies [89, 90, 91]. Here we selected furan, s‐tetrazine, pyrrole, imidazole, benzoquinone, acetone, formaldehyde, propanamide, acetamide, and pyridine. As shown in Figure 2a, excitation energies calculated at the LR‐PCM/M06‐2X/6‐31+G* level exactly match the $\omega_{0}^{\left(0I\right)}$ values because the $P_{\text{trans}}^{\left(0I\right)}$ is null: this results in a $R_{\text{solv}}\left(P_{\text{trans}}^{\left(0I\right)}\right)=0$ regardless of the solvent. Therefore, even for a solvent with low polarity, a SS approach is necessary to adequately describe solvation effects on $S_{0}\to T_{1}$ transitions. We highlight that for MRSF–TDDFT, implemented in the GAMESS code [64], only the LR–PCM solvation scheme is available. Since we proved that a LR‐PCM scheme does not provide a correction to singlet‐to‐triplet transitions, a flat horizontal line at $R_{\text{solv}}\left(P_{\text{trans}}^{\left(0I\right)}\right)=0$ is expected if one were to choose the coupling of this electronic structure approach with the LR‐PCM. As expected, VEM(UD) simulations give instead large corrections as the excited state density reorganization increases (i.e., for polar substrates whose excited triplets are then more stabilized by the presence of the solvent). Table S4 presents results for the entire benchmarking set.

Since accurately describing $T_{1}$ energies is key to modeling IST dyes, the calculations must include state‐specific polarization effects. Taking into account the discussion on electronic structure methods (see the previous section), we can therefore define a protocol for calculating $S_{1}$ and $T_{1}$ energies in the double‐hybrid TD‐DFT picture.

(3) $$\Delta E_{\left(\mathrm{solution}\right)}=\Delta E_{\mathrm{VEM}\left(\mathrm{UD}\right)}+\Delta E_{\mathrm{DH}}\;$$

Excitation energies obtained from Equation (3) properly incorporate SS description of the VEM(UD) term (obtained by the single‐hybrid analogue of B2PLYP) and a second‐order perturbative correction ($\Delta E_{\mathrm{DH}}$) on both the ground (MP2‐like) and the excited (CIS(D) like [70]) states: these two terms only depend on two‐electron integrals computed using MOs coming from a double‐hybrid (here B2PLYP) ground state PCM calculation. The definition presented in Equation (3) can be applied both to an adiabatic and a vertical description: if the right‐hand side terms of the equation are computed on ground state geometries, the energy is then referred to as vertical; if energies are obtained on excited state structures (i.e., VEM(UD)‐M062X‐def2‐TZVP) then we have moved to an adiabatic description. (Figure 2 and Table S6 show the application of this definition to the set of substrates of Figure 1a).

To refer back to the point made earlier on the performances of TD‐DFT, the weakness of the electronic structure method in the prediction of ΔE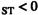 for systems **2**, **3**, and **4** does not allow one to draw unbiased conclusions on solvent effects for these substrates. Concerning systems **1** and **5**, the presence of a polarizable environment itself is insufficient to reverse the sign of the gap, but it does increase its value, contrary to what was suggested in a previous study [31]. However, it is fundamental to note that in Ref [31] the SS polarization response was not described by a self‐consistent and variational approach but only extrapolated in a perturbative framework using the refractive index of different solvents. Moreover, the SS solvation effects on excited‐state geometries were not considered. Indeed an essential role in determining the magnitude of ΔE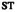 is played by the inclusion of adiabatic effects: system **1** exhibits a larger ΔE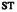 when energies are calculated on excited state structures and system **5** represents in this sense a limiting case since a negative gap is obtained only in the adiabatic picture. Nevertheless, we notice that there is not a unique tendency for the trend of $\Delta E_{\mathrm{ST}}$ gaps when moving from vertical to adiabatic energy differences: the behavior seems to depend on the specific system considered, despite their rigidity. Moving from a qualitative to a quantitative comparison with multireference simulations, we observe that for system **1**, the RMS–CASPT2 ΔE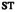 is larger than the VEM(UD) value, whereas for system **5**, the opposite trend is observed. The reason for this discrepancy can be again found in the electronic structure method, which yields a $\Delta E_{\mathrm{DH}}$ of −0.84 eV for system **5**, more than the twice as large as the $\Delta E_{\mathrm{DH}}=-0.35$ eV obtained for system **1**.

Excited‐state geometry effects on ΔE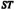 have already been investigated in a recent study by Jacquemin et al. [29] and are in complete agreement with our findings (see Figure 2 bottom left panel). As in previous EOM‐CCSD calculations in vacuo [29], heptazine presents a minimum on $S_{1}$ of $C_{3v}$ symmetry as a result of the puckering of the central nitrogen, while the minimum on $T_{1}$ exhibits an in‐plane deformation that leads to a $C_{s}$ geometry, as shown by UCCSD/cc‐pVDZ simulations as well [29].

Excited‐state properties are, of course, impacted by the choice of functional and solvation model [35, 48]. Table S7 reports that the switch of XC‐functional from M06‐2X to the single‐hybrid variant of B2PLYP produces non‐negligible effects on the optimized structures: these minima are generally more planar, with the central nitrogen atom lying closer to the plane than the structures obtained using M06‐2X. Using these latter results indeed leads to a closer agreement with coupled‐cluster values [29].

To summarize, when a double‐hybrid functional can properly describe a negative triplet‐to‐singlet gap, we propose using a protocol that consists of performing excited‐state optimizations at the VEM(UD) M06‐2X/def2‐TZVP level of theory and then simulating excitation energies on top of them using VEM(UD) double‐hybrid TD‐DFT calculations.

The results presented up to this point demonstrate that, potentially, a combination of a promising, quick, and accurate electronic structure method such as MRSF–TDDFT with a state–specific, self–consistent, and accurate solvation scheme such as VEM could match the accuracy and reliability of wavefunction methods that, unfortunately, are unfeasible for large systems. This can be achieved thanks to the inclusion of the static correlation in MRSF–TDDFT, complemented by dynamic correlation available by adopting a double–hybrid functional. However, at the moment, an implementation coupling MRSF–TDDFT and VEM is not available. While such an implementation is currently under development, we propose an alternative approach able to treat large molecules of experimental interest based on double‐hybrid TD‐DFT and VEM(UD) for energies and ES structures. Finally, for the sake of completeness, we point out that the VEM excitation energies can be computed by the NWChem software package [92] or, for Gaussian users, by the external tool VEM‐GAUSS implemented by Marenich, Cramer and Truhlar [93]. The availability of these tools enhances the applicability and accessibility of our proposed protocol, even if still the Lagrangian approach to include gradients is implemented only in local development versions of the Gaussian software by some of us [33].

### 3.3 Experimental Emitters: The B2PLYP/VEM(UD) Protocol at Work

**FIGURE 3:**
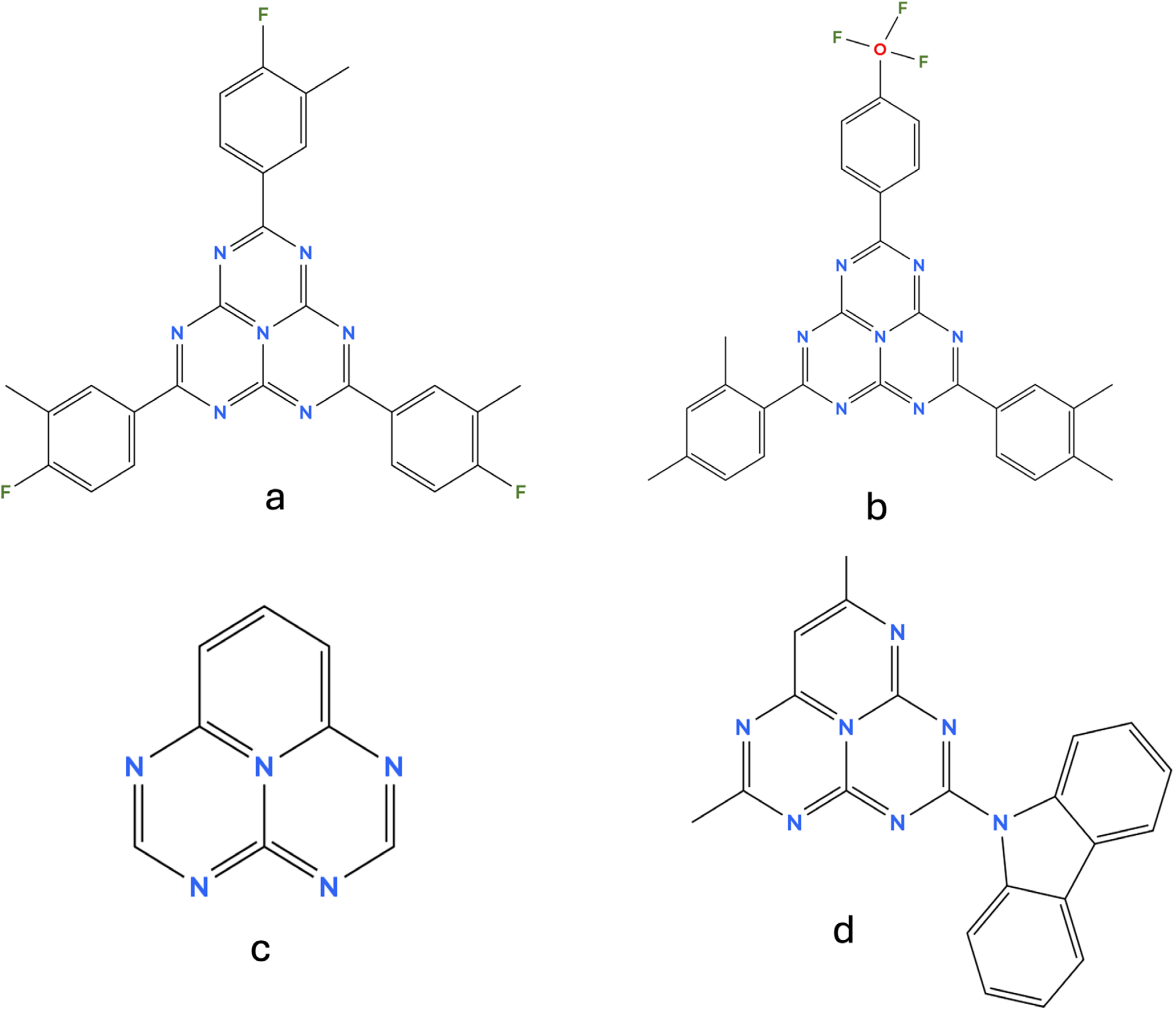
Structures of experimentally studied IST systems: HAP‐3MF (a), HzTFEX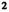 (b), 5AP (c), A6AP‐Cz (d).

Measurement of singlet‐triplet inversion is a non‐trivial task [79, 80, 81, 94] and this explains the limited amount of experimental literature, as well as the importance of having efficient computational screening methods [24, 30, 95]. Based on the good results obtained for heptazine with B2PLYP/VEM(UD), we employ our protocol for the simulation of larger substrates based on this core (see Figure 3), for which pioneering experimental evidence of a negative $\Delta E_{\mathrm{ST}}$ has been found [79, 80, 81, 94]. Moreover, increasing the system's dimension implies an augmented computational cost that can make difficult the simulation with CASPT2‐based approaches. We thus adopted MRSF–TD‐DFT simulations in combination with the M06–2X functional, which in Table 1 we demonstrated to be a suitable alternative. In some cases we encountered convergence problems with M06–2X, and we switched to BH&HLYP as the standard in SF methods. Except for A6AP–Cz [80], we obtained $\Delta E_{\mathrm{ST}}<0$ for all systems, supporting experimental evidence and confirming the suitability of MRSF–TD‐DFT in simulating this property. However, since only LR‐PCM solvation is available for MRSF–TD‐DFT, we will analyze the effects of a state–specific solvation model applying our B2PLYP/VEM(UD) protocol.

The heptazine‐based OLED chromophore HAP‐3MF, shown in (Figure 3a), is generally regarded as the first inverted singlet‐triplet emitter implemented in OLEDs [96]. Since the transition to the first excited singlet possesses a very weak oscillator strength, regardless of the embedding and electronic structure description, one can argue that higher singlet excited states are populated under optical excitation and decay to $S_{1}$ through non‐radiative mechanisms. As per Kasha's rule, $S_{1}$ is then involved in radiative and non‐radiative processes that characterize IST emitters. This substrate experimentally exhibited a negative $\Delta E_{\mathrm{ST}}$ in toluene (−220 meV) and in acetonitrile (−190 meV) based on an investigation of its fluorescence and phosphorescence spectra [94].

Consistently with our benchmarks, our protocol demonstrates strong reliability for the prediction of singlet‐triplet inversion in environments of low polarity. As shown in Table 2, we are able to quantitatively reproduce the experimental values of $\Delta E_{\mathrm{ST}}$ in toluene (i.e., −270 meV w.r.t. −220 meV) for HAP‐3MF. The absolute value of $\Delta E_{\mathrm{ST}}$ increases in passing from the vertical to the adiabatic picture. Table 2 also shows the emissive and non‐radiative rates of HAP‐3MF (see also Table S8), including vibronic effects. The calculated values are consistent with experimental estimates [94, 97] as well as previous simulations performed in vacuo [73] at DFT/MRCI level. Indeed, Li et al. [97] estimated a $k_{f}$ of $1\times 10^{6}\;s^{-1}$ based on the measured fluorescence lifetime, in complete agreement with our prediction. Concerning non‐radiative decays, our calculations point out that $k_{\mathrm{ISC}}>k_{\mathrm{IC}}>k_{\text{rISC}}$: this seems to confirm the experimental hint of a probable $k_{\text{rISC}}<k_{\mathrm{ISC}}$ due to the measured phosphorescence emission that depopulates radiatively the $T_{1}$ electronic state [94]. The second heptazine‐analogue system taken into account is HzTFEX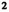 (see Figure 3b and Figure S1), which shows a slight negative $\Delta E_{\mathrm{ST}}$ of −11 ± 2 meV, estimated in a deaerated toluene solution, as reported in Ref. [79]. In the same study, the singlet‐triplet inversion was validated by gas‐phase simulations employing ADC(2) and CASPT2 (12,12) with a double‐zeta basis set, that predict an inversion of −34 and −184 meV, respectively.

**TABLE 2:** $\Delta E_{\mathrm{ST}}$ (in meV, top) and radiative/non‐radiative transition rates (in $s^{-1}$, bottom).

System	Adiabatic MRSF–TDDFT/def2–TZVP C–PCM	VEM(UD)‐B2PLYP vertical	VEM(UD)‐B2PLYP adiabatic
HAP‐3MF	−122 ^a^	−215	−270
HzTFEX 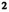	−114 ^a^	15	−336
5AP	−5 ^b^	244	−125
A6AP‐Cz	225 ^b^	−381	403

VEM(UD)‐B2PLYP radiative and non‐radiative transition rates ($s^{-1}$)					
System	Temperature (K)	$k_{F}$	$k_{\mathrm{IC}}$	$k_{\mathrm{ISC}}$	$k_{\text{rISC}}$
HAP‐3MF	300	$1.0\times 10^{6}$	$5.0\times 10^{4}$	$5.0\times 10^{5}$	$5.8\times 10^{4}$
HzTFEX 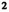	300	$2.8\times 10^{7}$	$1.6\times 10^{9}$	$9.9\times 10^{5}$	$1.9\times 10^{6}$
5AP	20	$1.2\times 10^{5}$	$9.3\times 10^{11}$	$1.6\times 10^{4}$	$1.8\times 10^{3}$
A6AP‐Cz	300	$1.1\times 10^{4}$	$1.5\times 10^{11}$	$1.3\times 10^{5}$	$8.6\times 10^{4}$
A6AP‐Cz (corrected for vertical energies)	300	$5.4\times 10^{4}$	$1.8\times 10^{11}$	$1.5\times 10^{5}$	$3.5\times 10^{5}$

^a^ M06–2X functional.

^b^ BH&HLYP functional.

Table 2 reports the B2PLYP/VEM(UD) results pinpointing the importance of including adiabatic effects: the $\Delta E_{\mathrm{ST}}$ gap becomes negative and of the same magnitude as determined for HAP‐3MF, in analogy with what is observed for the gas‐phase CASPT2 calculations [79]. The primary effect is attributed to the distortion of the heptazinic core at the S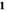 minimum, which belongs to the C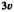 point group, in analogy to the pure heptazine case previously discussed. Moving to emission and non‐radiative terms, we computed a $k_{f}$ value in close agreement with the experimental prompt fluorescence constant of $7.1\times 10^{7}\;s^{-1}$ [79]. and the B2PLYP/VEM(UD) simulations effectively reproduce $k_{\text{rISC}}>k_{\mathrm{ISC}}$, and their ratio (1.8 experimentally [79], in close agreement with the simulated ratio of 1.9). Finally, it is important to point out that both direct and reverse ISC rate values have been obtained by fitting the experimental photo‐luminescence (PL) spectra registered in PPF polymer host matrix under a N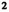 atmosphere and not in solution: [79] and this probably explain why the experimental absolute values of nonradiative rates are of one order of magnitude ($k_{\text{rISC}}=4.2\times 10^{7}\;s^{-1}$ and $k_{\mathrm{ISC}}=2.3\times 10^{7}\;s^{-1}$) larger than the computed ones in toluene. 1,3,4,6,9b‐pentaazaphenalene, herein referred to as 5AP, is the third substrate examined in this part of the study. Wilson et al. [81] recently measured via high‐resolution cryogenic anion photoelectron spectroscopy a $\Delta E_{\mathrm{ST}}=-0.047$ eV for this substrate. The same authors performed a comprehensive analysis of the computational predictions of IST available in the literature [29, 51, 98] and conducted their own simulations on the chromophore. EOM‐CCSD(T) with triple‐zeta basis sets calculations indeed yield values with the highest agreement to the experimental measure, while ADC(2), CC3, and CC2 generally provide more negative $\Delta E_{\mathrm{ST}}$ (i.e., of the order of −100 meV). These values are consistent with our estimated VEM(UD) ST gap of −125 meV. The 5AP core exhibits a very weak S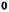 → $S_{1}$ oscillator strength, which significantly limits the fluorescence quantum yield; therefore, no experimental measurements of radiative and non‐radiative rates have been reported. Given that this system can be functionalized with different substituent groups to improve its spectroscopic properties [99, 100], the possibility of making a direct comparison of the VEM(UD) performances with experimental data on the core is particularly valuable. The fourth and final substrate we investigated is A6AP‐Cz: in a recent study, ΔE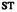 values of −45 meV and −73 meV were measured using two different spectroscopic techniques [80]. The same authors predicted the vertical ΔE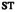 of the compound in vacuo using the double‐hybrid functional B2PLYP and def2‐TZVP basis set and obtained a negative value of =−44 meV, but estimates of the adiabatic gap were not made. The adiabatic singlet–triplet gap computed using our VEM(UD) protocol is positive and, in contrast to all other systems, ΔE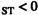 is predicted only when considering the difference between the vertical excitation energies. A similar trend is observed in simulations performed at the MRSF‐TDDFT level of theory. This finding is consistent with the results reported by Okumura et al. [80], but also underscores the need for a more comprehensive investigation of the system. Vertical $\Delta E_{\mathrm{ST}}$s were used in the calculation of radiative and non‐radiative rates: we correctly reproduce the experimental observation of a large non‐radiative decay constant from S_1_ to S_0_ which significantly affects the quantum yield of the system.

## 4 Conclusions

This work assessed various computational strategies for calculating singlet–triplet gaps, including the proper treatment of the electronic correlation and state‐specific solvent polarization effects. In the first part, we used multireference calculations as a benchmark to demonstrate when one can recover electronic correlation in the TD‐DFT picture, thereby determining negative $\Delta E_{\mathrm{ST}}$. The MRSF approach can treat doubly excited configurations, recovering static effects on top of TD‐DFT treatment. Instead, double‐hybrid functionals only account for dynamic correlation, allowing us to predict $\Delta E_{\mathrm{ST}}<0$ only for specific substrates. In the second part, we described solvation effects on both excited singlet and triplet states. We first highlighted the significant limitations of the LR‐PCM model for studying singlet‐to‐triplet transitions and emphasized the importance of accurately describing the solvent response to variations in the solute's polarization with a state‐specific model. Thanks to the accurate VEM(UD) approach to TD‐DFT in solution, which is state‐specific, self‐consistent, and variational, we showed that interactions with the environment can increase the absolute value of negative $\Delta E_{\mathrm{ST}}$ but are not able to revert the sign of the gap for systems that present $\Delta E_{\mathrm{ST}}>0$ *in vacuo*. Finally, we employed an effective protocol for simulating substrates based on the heptazinic core, for which negative $\Delta E_{\mathrm{ST}}$ gaps have been experimentally measured. The B2PLYP/VEM(UD) protocol here presented is not only able to reproduce $\Delta E_{\mathrm{ST}}<0$, but also provides emissive and non‐radiative transition rates in very good accordance with experimental estimates. A final point deserves here a comment: in principle both state‐specific medium polarization and dispersion interactions should be included to properly treat the complex and retarded response of a polarizable bath (the environment) to a perturbation on a quantum system (the solute), as rooted in the theory of open quantum systems [101]. Depending on the expression used for dispersion, LR‐PCM energy can recover part of the solute‐solvent dispersion interaction [101, 102, 103, 104], and some of us recently proposed a cLR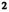 method [34]. However, we could not use this approach here because the LR‐PCM terms are null for singlet‐to‐triplet excitations, as previously stated; therefore, the dispersion term cannot be approximated for these transitions but only for singlets by a cLR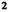 treatment. Formulating a rigorous approach that accounts for the contribution of dispersion to both singlet and triplet excited states proves to be a complicated task. An empirical correction was derived based on solute polarizability and solvent refractive index (proposed by Marenich, Cramer, and Truhlar [105]), but it was parameterized solely for singlet excited states and could not be readily extended to triplets, which are crucial for the scope of our study. Recently, Amovilli and Floris proposed a method based on variational quantum Monte Carlo in PCM [106], but the test includes only singlets, and it has not yet been implemented in any available code. We therefore decided not to include dispersion effects in this study, but work is in progress to account for all those effects, in an open quantum system framework and using a non‐Markovian stochastic Schrödinger equation approach [101].

## Conflicts of Interest

The authors declare no conflicts of interest.

## Supporting information


**Data S1:** jcc70267‐sup‐0001‐supinfo.pdf.

## Data Availability

The data that supports the findings of this study are available in the Supporting Information of this article.
