# Supplementary material for: On the Role of Electronic Correlation and State‐Specific Environment Polarization in Singlet–Triplet Gap Inversion
Source: J Comput Chem. 2025 Nov 11;46(30):e70267. doi: 10.1002/jcc.70267 (PMC12604461; doi:10.1002/jcc.70267)
Supplement: Supplementary file 1 — Data S1: jcc70267‐sup‐0001‐supinfo.pdf. [file JCC-46-0-s001.pdf]

On the role of electronic correlation and state-specific environment polarization in Singlet–Triplet gap inversion

Ester Salvi,[a] Giacomo Agostini,[b] Simone Veglianti,[b] Gustavo Juliani Costa,[a] Luca De Vico,[b] Daniele Padula,[b*] and Ciro A. Guido[a†]

October 17, 2025

Supporting Information

*To whom correspondence should be addressed. Email: daniele.padula@unisi.it
†To whom correspondence should be addressed. Email: ciro.guido@uniupo.it

Contents

List of Figures

S1 Views of the (VEM)-PCM/(TD)-DFT/M06-2X/def2-TZVP $HzTFEX_2$ structures: (left) ground state minimum, (right) S_1 state minimum. S6

List of Tables

S1 RMSD in terms of bond lengths, bond angles, dihedrals, and overall molecular geometry between RMS–CASPT2/ANO–R2 and TDDFT/M06–2X/def2–TZVP S_1 equilibrium geometries. S3
S2 Adiabatic ΔE_{ST} (in meV) computed with MRSF–TD-DFT/def2–TZVP on TD-DFT/M06–2X/def2–TZVP geometries. S3
S3 Adiabatic ΔE_{ST} (in meV) computed with MRSF–TD-DFT/def2–TZVP, including solvation effects (LR-CPCM) for toluene, on TD-DFT/M06–2X/def2–TZVP geometries. S3
S4 Vertical excitation energies at LR–TDDFT/M06–2X/6–31+G* S4
S5 LR–TDDFT/M06–2X/def2–TZVP NTO character and metrics S4
S6 Benchmarking of VEM(UD)-B2PLYP adiabatic and vertical ΔE_{ST} against RMS-CASPT2/ANO-R2 SS-PCM ΔE_{ST} (all in meV). S5
S7 Effect of XC-correlation functional excited state (TD)-DFT geometrical parameters: dihedral angle (deg) and bond lenght (Å) S5
S8 HAP–3MF dipole strengths for the first and second singlet excited state at B2PLYP/def2–SVPP and B2PLYP/def2–TZVP (all in au) S5

Table S1: RMSD in terms of bond lengths, bond angles, dihedrals, and overall molecular geometry between RMS–CASPT2/ANO–R2 and TDDFT/M06–2X/def2–TZVP S_1 equilibrium geometries.

Molecule	Active Space	bonds / Å	angles / deg	dihedrals / deg	RMSD / Å
1	(14,13)	0.009	0.682	2.207	0.023
2	(14,13)	0.005	0.145	0.006	0.010
3	(12,13)	0.006	0.220	0.010	0.010
4	(14,11)	0.006	0.415	1.686	0.015

Table S2: Adiabatic ΔE_{ST} (in meV) computed with MRSF–TD-DFT/def2–TZVP on TD-DFT/M06–2X/def2–TZVP geometries.

**Molecule**	**BH&HLYP**	**PBE0**	**CAM–B3LYP**	**B2PLYP**
1	-117	4	-73	-139
2	-71	16	-58	-87
3	-98	5	-64	-116
4	110	82	91	112
5	-37	20	-17	-49

Table S3: Adiabatic ΔE_{ST} (in meV) computed with MRSF–TD-DFT/def2–TZVP, including solvation effects (LR-CPCM) for toluene, on TD-DFT/M06–2X/def2–TZVP geometries.

**Molecule**	**BH&HLYP**	**M06–2X**	**PBE0**	**CAM–B3LYP**	**B2PLYP**
1	−113	−158	9	−67	−135
2	−71	−98	15	−58	−86
3	−94	−140	7	−62	−112
4	123	107	86	97	122
5	−34	−64	21	−16	−46

Table S4: Vertical S_1 and T_1 energies at TD-DFT/M06-2X/6-31+G*

TOL						
**molecule**	S$_1$			T$_1$		
	ω_0	LR-PCM	VEM-PCM	ω_0	LR-PCM	VEM-PCM
acetone	4.151	4.147	4.093	3.688	3.688	3.629
piridina	5.012	5.002	4.877	4.400	4.400	4.270
acetamide	5.555	5.552	5.446	5.126	5.126	5.018
benzochinone	2.703	2.702	2.680	2.303	2.303	2.279
formaldeide	3.770	3.764	3.706	3.238	3.238	3.175
furano	6.732	6.627	6.723	4.391	4.391	4.382
imidazolo	7.016	6.997	6.820	4.950	4.950	4.930
pirrolo	7.024	6.921	7.013	4.756	4.756	4.743
propanamide	5.608	5.606	5.503	5.209	5.209	5.105
stetrazina	2.257	2.254	2.253	1.664	1.664	1.652

Table S5: NTO character and metrics of $S_0 \rightarrow S_1$ and $S_0 \rightarrow T_1$ transitions at LR–TDDFT/M06–2X/def2–TZVP

Heptazine					
Solvent (VEM-PCM)	$\vec{S}$	$\Phi_i^{'}$	$\Phi_a^{'}$	λ_{ia}	NTO $\Delta r(\AA)$
TOL	0	44	45	0.97943	0.01052
TOL	1	44	45	0.97327	0.01782
ACN	0	44	45	0.97831	0.01032
ACN	1	44	45	0.97191	0.01735
**Cyclazine**					
Solvent (VEM-PCM)	$\vec{S}$	$\Phi_i^{'}$	$\Phi_a^{'}$	λ_{ia}	NTO $\Delta r(\AA)$
TOL	0	44	45	0.99762	0.00389
TOL	1	44	45	0.99754	0.00559
ACN	0	44	45	0.99757	0.00374
ACN	1	44	45	0.99774	0.00557

Table S6: Benchmarking of VEM(UD)-B2PLYP adiabatic and vertical ΔE_{ST} against RMS-CASPT2/ANO-R2 SS-PCM ΔE_{ST} (all in meV).

**Molecule**	**B2PLYP/def2-TZVP-VEM(UD)**		**RMS-CASPT2/ANO-R2 SS-PCM**
	Adiabatic	*Vertical*	*Adiabatic*
1	−38	−19	−185
2	72	69	−54
3	38	0	−124
4	79	218	−5
5	−415	52	−43

Table S7: Effect of XC-correlation functional excited state (TD)-DFT geometrical parameters: dihedral angle (deg) and bond lenght (Å)

	M06-2x/def2-TZVP			BLYP53/def2-TZVP		
			**Heptazine core dihedral**			
**state**	*in vacuo*	LR-PCM TOL	VEM-PCM TOL	*in vacuo*	LR-PCM TOL	VEM-PCM TOL
S_1	-4.5	-3.7	-4.5	-3.8	-3.4	-3.6
T_1	-1.8	0.0	0.0	0.0	0.0	0.0
			**Heptazine core C-N bond**			
**state**	*in vacuo*	LR-PCM TOL	VEM-PCM TOL	*in vacuo*	LR-PCM TOL	VEM-PCM TOL
S_1	1.417	1.410	1.415	1.408	1.405	1.406
T_1	1.408	1.405	1.405	1.413	1.399	1.399

Table S8: HAP–3MF dipole strengths for the first and second singlet excited state at B2PLYP/def2–SVPP and B2PLYP/def2–TZVP (all in au)

	Dipole strenghts		Oscillator strenghts	
State	def2-SVPP	def2-TZVP	def2-SVPP	def2-TZVP
S_1 GAS	0.0007	0.0009	0.0001	0.0001
S_1 TOL	0.0012	0.0015	0.0001	0.0001
S_2 GAS	10.5204	10.6764	1.1330	1.1537
S_2 TOL	13.0558	13.3234	1.3747	1.4044

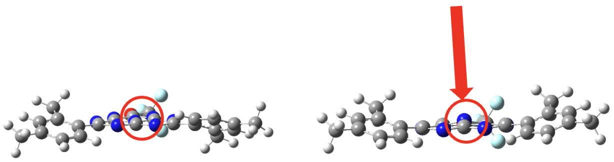

Fig. S1: Views of the (VEM)-PCM/(TD)-DFT/M06-2X/def2-TZVP $HzTFEX_2$ structures: (left) ground state minimum, (right) S_1 state minimum.
